# Relating Fresh Concrete Viscosity Measurements from Different Rheometers

**DOI:** 10.6028/jres.108.021

**Published:** 2003-06-01

**Authors:** Chiara F. Ferraris, Nicos S. Martys

**Affiliations:** National Institute of Standards and Technology, Gaithersburg MD 20899-0001

**Keywords:** concrete rheometers, dissipative particle dynamics modeling, plastic viscosity, rheology

## Abstract

Concrete rheological properties need to be properly measured and predicted in order to characterize the workability of fresh concrete, including special concretes such as self-consolidating concrete (SCC). It was shown by a round-robin test held in 2000 [1,2] that different rheometer designs gave different values of viscosity for the same concrete. While empirical correlation between different rheometers was possible, for a procedure that is supposed to “scientifically” improve on the empirical slump tests, this situation is unsatisfactory. To remedy this situation, a new interpretation of the data was developed. In this paper, it is shown that all instruments tested could be directly and quantitatively compared in terms of relative plastic viscosity instead of the plastic viscosity alone. This should eventually allow the measurements from various rheometer designs to be directly calibrated against known standards of plastic viscosity, putting concrete rheometry and concrete workability on a sounder materials science basis.

## 1. Introduction

In the concrete industry, workability is defined as “the ease and homogeneity for which the concrete or mortar can be placed, consolidated and finished” [3]. Ideally, concrete workability should be characterized by its rheological properties, thus establishing a materials science basis. These properties are usually defined as the Bingham parameters: yield stress and plastic viscosity [4]. It has been shown that the most common workability test used, the slump cone test (ASTM C443) [5], correlates well only with the yield stress [6]. There are no standard tests of fresh concrete that relate directly to the plastic viscosity. Hence, the workability of concrete is not completely measured or specified by current standard tests.

In response to this fact, at least five concrete rheometers have been designed to measure both the yield stress and plastic viscosity of concrete. These rheometers were compared in 2000 during a round-robin test [1]. It was found that although good empirical correlations could be found between the rheometers, the absolute values of the rheological parameters depended on the instrument used. As a result, the concrete industry is unable to specify workability in terms of rheological properties, because the plastic viscosity cannot be easily and uniquely measured. Therefore, a different approach to compare the results from various rheometers has become necessary.

The new approach presented here uses the relative plastic viscosity instead of the plastic viscosity. The relative plastic viscosity of a suspension is defined as the ratio of the plastic viscosity of the whole suspension to the plastic viscosity of the embedding fluid matrix or medium. In concrete, the embedding matrix can be defined as the mortar, while in mortar the matrix would be the cement paste. The inclusions or particles in the suspension are the coarse aggregates in concrete or the sand in mortar. Therefore, the relative plastic viscosity of a concrete is the plastic viscosity of the concrete divided by the plastic viscosity of the mortar. It is assumed that the mortar used to determine the plastic viscosity of the matrix has the same composition as the mortar in the concrete. The relative plastic viscosity is a function of the concentration of the particles and their shape. Thus, for a given concrete, a plot could be prepared comparing the relative plastic viscosity and the coarse aggregate concentration. It will be shown that the relative plastic viscosity does not depend on the rotational rheometer used. If all data can really be plotted on the same curve using the relative plastic viscosity, it would allow direct comparisons of the data from all rheometers, which until now has not been possible.

In this study, the relative plastic viscosities of several concrete mixes were determined by using computer simulation, two different concrete rheometers, and a parallel plate cement paste/mortar rheometer. By plotting all the data on a graph of the relative plastic viscosity versus the concentration of particles (i.e., coarse aggregates), it can be shown that the main influence on the relative plastic viscosity is the aggregate concentration (although other factors such as shape could play a role). The data set used in this paper is small, therefore our observation should be confirmed with further testing. A second round-robin test comparing concrete rheometers is being planned by American Concrete Institute (ACI) committee 236A in 2003. The results will be used to further improve this method.

## 2. Theoretical Approach

Most rotational rheometers are based on the principle that the material is stirred at a controlled speed and the resulting torque is measured. In the case of a Newtonian fluid, the viscosity is defined as the ratio between the stress and the shear rate [7]. Concrete and mortar are generally accepted to be Bingham fluids [6]. In such materials, the plastic viscosity is defined as the slope of the stress versus shear rate in the high shear rate limit. Most rheometers measure torque versus rotational speed. Therefore to obtain the true or absolute plastic viscosity, the slope of the curve should be corrected by a function, *f*, that depends on the rheometer geometry and experimental conditions. So the following equation could be used:

$$\frac{\Delta T}{\Delta V}=\eta_{T}\cdot f(G,C)$$ (1)

where

Δ*T*/Δ*V* = Slope of the torque (*T*) versus rotational speed (*V*)
*η*_T_ = True or absolute plastic viscosity
*f* (*G*,*C*) = function depending on the rheometer geometry (*G*) and experimental conditions (*C*).

The function *f* is not fully known for most of the concrete rheometers due to their complex geometry and the lack of a standard material that could be used for calibration. Oils are often used as standard materials but they are too expensive and have a viscosity too low to be used in a large concrete rheometer. These oils are designed for small rheometers such as the one used for cement paste. The parameters, *G* and *C*, of the function, *f*, take into account not only the type of rheometer (parallel plate or coaxial) but also the type of coupling between the fluid and the rheometer, the type of fluid tested, environmental conditions and the limits of the instrument (the limits of measurable torque or rotational speed). It could be imagined that the parameters, *G* and *C*, vary with the type of fluid used in the same rheometer. However, as will be shown below by the experimental results, the factor *f* (*G*,*C*) depends more on the type of rheometer than on the type of fluid tested. This observation should be further confirmed by more testing. Due to the lack of knowledge of the function *f*, the true or absolute plastic viscosity cannot be known with low uncertainty. This could explain why it was not possible to compare the absolute values of the plastic viscosity obtained with the concrete rheometer during the round robin test [1,2].

A method should be developed to either determine this function *f* or to eliminate it. Suppose that two measurements are performed with the same rheometer on two different mixtures (1) and (2). The following equation could be written:

ΔT1ΔV1ΔT2ΔV2=ηT1⋅f(G,C)ηT2⋅f(G,C)=ηT1ηT2 (2)

where the indices (1) and (2) stand for the two mixtures tested in the same rheometer. For instance, material 1 could be the concrete while material 2 could the mortar with the same composition of the concrete without the coarse aggregates. This ratio, *η*_T1_/*η*_T2_, is defined as the relative plastic viscosity.

From Eq. (2), we could say that the relative plastic viscosity does not depend on the rheometer used. This implies that plots of the relative plastic viscosity, measured with different rheometers, versus a mixture factor, such as the coarse aggregate concentration, should all be on one curve. It also implies that the relative plastic viscosity is independent of the physical units used to represent plastic viscosity. This hypothesis was tested using a wide variety of mixtures, although more types of rheometers should be included to confirm this finding.

## 3. Data Used

**Fig. 1:**
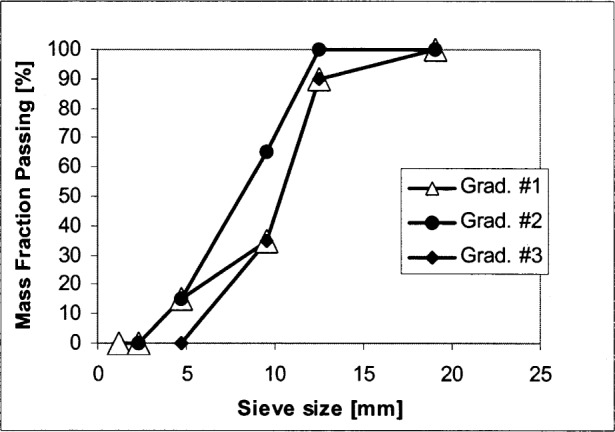
Aggregate gradations used for the computer simulations shown in Fig. 2.

To determine if the relative plastic viscosity could be used to compare the data from different rheometers and/or computer simulations, we examined the results of four sets of data:

- A coaxial rheometer (BML^1^ [8]) with a high range water reducer admixture (HRWRA) (Table 1).
- A vane rheometer (IBB [9]) in which three concrete mixes were prepared with different air contents (Table 1) and different coarse aggregate concentrations.
- A computer simulation (see description below) in which three types of spherical aggregate gradation were used. The distributions used are shown in Fig. 1.
- A parallel plate rheometer designed for cement paste and mortar in which various concentrations of monosized glass beads were added to cement paste.

The detailed description of the BML and IBB rheometers can be found in various publications [8,9]. These were two of the rheometers used in the international round-robin tests [1]. Table 1 shows the composition of the mixes used. It should be noted that the plastic viscosity measured with the IBB is not given in fundamental units of Pa s but in Nm s. Therefore, it is impossible to directly compare the results from the two rheometers. Nevertheless, an empirical correlation function was determined for each pair of rheometers as described in Ref. [1].

Several simulations of hard sphere systems [10,11], imbedded in an isothermal Newtonian fluid, were carried out where the size distribution of the spheres was consistent with those shown in Fig. 1. The number of spheres varied from about 200 to 500 depending on the solid fraction. By applying a constant strain to this system a shear flow developed. Sphere movements and sphere interaction were modeled using a method based on dissipative particle dynamics (DPD) [12]. The viscosity was then determined from calculation of the averaged stresses for a given strain rate [12].

The paste measurements were conducted using a parallel plate rheometer used for cement paste [13]. This rheometer was modified from the description in Ref. [13] to accommodate mortar. The plates were 60 mm in diameter (instead of the 35 mm diameter usually used for cement paste) and a confinement ring was used. This ring has an internal diameter of 62 mm and a height of about 20 mm. The gap between the two plates was 10 mm for both the cement paste and mortar mixtures. The cement paste was prepared using a Type I cement and a *w*/*c* ratio of 0.45, with no admixtures. The glass beads were nominally 1 mm in diameter and the volume concentration was varied from 0 % to 50 %. This type of aggregate was selected to provide validation data for the DPD model as they were mono-dispersed and spherical and thus straight forward to simulate.

## 4. Discussion

The relative plastic viscosity was calculated for all mixtures by dividing the plastic viscosity of the mixture containing the coarse aggregates or particles with the plastic viscosity of the matrix (mortar or cement paste). Care was taken to ensure that the matrix that was measured alone was identical to the matrix in the mixture.

**Fig. 2:**
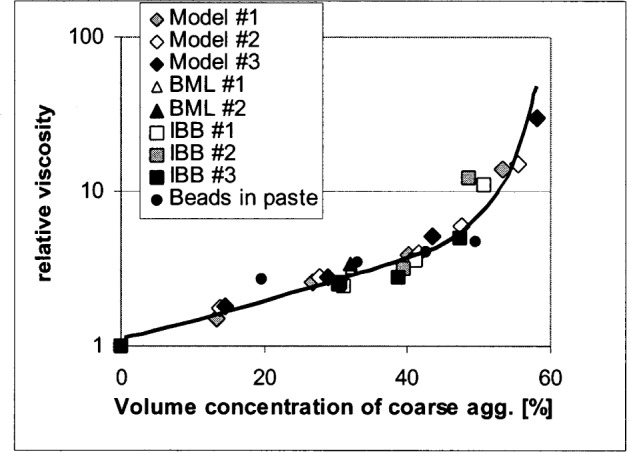
Relative plastic viscosity as a function of the particle concentration. The composition of the mixtures is given in Table 1. The three model series correspond to the three gradations used in Fig. 1. No error bars are shown because these are the results of only one set of data (no replica). The solid line is simply a guide for the eye.

All the relative plastic viscosities measured are plotted in Fig. 2. It can be seen that all data are approximately on the same curve. It should be pointed out that the geometry of the various rheometers were not the same and also that the absolute values of the plastic viscosity are not even expressed in the same units in some cases (i.e., IBB). This is explained by Eqs. (1) and (2) and the related discussion. The relative plastic viscosity eliminates the correction factor as seen in Eq. (2). At this point, we do not know the uncertainty of the data shown in Fig. 2 because there was only one trial at each of the data points presented. This is an area that will be further investigated.

Obviously, it is expected that varying the shapes of aggregate would generate a family of curves (relative viscosity versus aggregate concentration) similar to the one shown in Fig. 2. This statement should be confirmed by acquiring more data with different mixture designs, aggregate shape and size distributions, and other rheometers. Assuming that this finding is true, the following scenarios could be imagined for quantitatively comparing rheometers:

First, if a mortar is measured using a rheometer that could be calibrated, using a standard oil for example, all plastic viscosity values could be corrected using a factor (*CF*) that is the ratio between the mortar plastic viscosity measured with the calibrated rheometer (*η*_m1_) and with the concrete rheometer (*η*_m2_). The correction factor will be:

$$CF=\eta_{m1}/\eta_{m2}$$ (3)

This correction factor does not depend on the condition that all relative viscosities fall on one curve. On the other hand, in order to compare concrete viscosities, it is necessary to examine the relative plastic viscosity, because the *CF* factor cannot be obtained for concrete, as there are no calibrated concrete rheometers. From Eq. (2) we can state that the relative plastic viscosity is independent of the rheometer or units used for the measurement. Figure 2 shows that the relative plastic viscosity of concrete does not depend strongly on the rheometer used but rather mainly on the concentration of coarse aggregates. Therefore, the following equation could be written:

$$\frac{\eta_{\text{Tc}}}{\eta_{\text{Tm}}}=\frac{\eta_{c}}{\eta_{m}}$$ (4)

where

*η*_m_ is the as-measured plastic viscosity of the matrix or mortar
*η*_c_ is the as-measured plastic viscosity of the concrete
*η*_Tm_ is the true or absolute plastic viscosity of the matrix or mortar
*η*_Tc_ is the true or absolute plastic viscosity of the concrete

From Eqs. (3) and (4), we can calculate *η*_Tc_:

$$\eta_{\text{Tc}}=\frac{\eta_{c}}{\eta_{m}}\cdot \eta_{\text{Tm}}=\frac{\eta_{c}}{\eta_{m}}\cdot CF\cdot \eta_{m}=CF\cdot \eta_{c}$$ (5)

Therefore, the true value of the plastic viscosity of a concrete can be calculated from Eq. (5). Note that Eq. (5) is definitely dependent on the validity of Eq. (4), while Eq. (3) is not at all dependent on Eq. (4)

Second, if a calibrated rheometer is not available and the goal is to simply compare the as-measured viscosities from two or more rheometers, one of the rheometers could be used as a “reference”, and one could then proceed with the same calculation as presented above.

Finally, it is obvious that it might not always be necessary to calculate the absolute value of the concrete plastic viscosity. Different concrete mixtures could simply be compared using the relative plastic viscosity alone. This will allow the comparison of measurements obtained from various rheometers even if the plastic viscosity results were not in the same units.

The procedure based on Eq. (4) depends on the observation that all relative plastic viscosities versus aggregate concentration are on the same curve. It is possible that factors that were not considered here might intervene, such as the coupling of the walls with the coarse aggregates. The significance of the variation needs to be established by conducting more measurements. Further data need to be collected to definitively establish the existence of a master curve relating relative plastic viscosity with coarse aggregate concentration, shape, or other factors.

In conclusion, it has been shown for the rheometers used that the relative plastic viscosity does not seem to depend on the rheometer but only on the amount of coarse aggregate (or particle) that were added to the matrix (mortar or cement paste). Therefore, the relative plastic viscosity can be used to compare data from various instruments even when a calibration with a standard material is not available and the results from the rheometers are given in different units. Some tests of mortar will be included in phase II of the ACI sponsored round-robin tests of four commercially available concrete rheometers to be held in 2003, in order to further test the validity of this method. Another implication of this conclusion is that modeling of the flow of concrete can be reduced to the flow of particles in a matrix. The only variable to be modified is the shape and concentration of the particles or aggregates. If the relative plastic viscosity is given and the mortar plastic viscosity is measured, the plastic viscosity of the concrete can be calculated. This procedure is being developed at NIST by creating a database searchable by the shape and the gradation of the coarse aggregates. The data will be presented as a curve of relative plastic viscosity versus the concentration of the coarse aggregates [14]. This method of presenting the data related to plastic viscosity will allow a leap forward in the interpretation of the data provided by various concrete rheometers, which will eventually allow optimization of concrete workability in terms of the materials used and for the desired performance.

## Figures and Tables

**Table 1:** Mix designs for the mortars tested using BML and IBB rheometers. The coarse aggregate concentration was varied (see Fig. 2)

Mix designation for Fig. 2	Rheometer used	Water/Cement Mass fraction	Sand/Cement Mass fraction	Air Entrainer (mL/100 kg of cement)
IBB #1	IBB	0.50	1.98	none
IBB #2	IBB	0.50	1.98	26.0
IBB #3	IBB	0.50	1.98	65.2
BML #1^a^	BML	0.38	2.00	none
BML #2^a^	BML	0.38	2.00	none

a The same dosage (26 mL/100kg) of two different high range water reducers was used in these two mixes.

## Footnotes

1 Commercial equipment, instruments, and materials mentioned in this paper are identified to foster understanding. Such identification does not imply recommendation or endorsement by the National Institute of Standards and Technology (NIST), nor does it imply that the materials or equipment identified are necessarily the best available for the purpose.

## Glossary

Δ*T*/Δ*V*: = Slope of the torque (*T*) versus rotational speed (*V*)
*η*_T_: = True or absolute plastic viscosity
*f* (*G*,*C*): = function depending on the rheometer geometry (*G*) and experimental conditions (*C*)
*CF*: = Correction Factor
*η*_mi_: = mortar plastic viscosity measured with the calibrated rheometer *i*
*η*_m_: = as-measured plastic viscosity of the matrix or mortar
*η*_Tm_: = true or absolute plastic viscosity of the matrix or mortar
*η*_c_: = as-measured plastic viscosity of the concrete
*η*_Tc_: = the true or absolute plastic viscosity of the concrete
